# Translation, Transcultural Adaptation, and Validation of the Brazilian Portuguese Version of the Body Image Disturbance Questionnaire for Scoliosis (BR-BIDQ-S)

**DOI:** 10.1055/s-0044-1785463

**Published:** 2024-04-10

**Authors:** Marcos Almeida Matos, Maria Clara Freitas, Rony Britto Fernandes, Luís Fernando Weber de Oliveira, Robert Meves

**Affiliations:** 1 Escola Bahiana de Medicina e Saúde Pública, Salvador, BA, Brasil; 2 Universidade do Estado da Bahia, Salvador, BA, Brasil; 3 Departamento de Ortopedia e Traumatologia, Programa de Coluna Vertebral da Santa Casa da Bahia, Hospital Santa Izabel, Salvador, BA, Brasil; 4 Grupo de Coluna do Departamento de Ortopedia e Traumatologia (DOT), Santa Casa de SP, Pavilhão Fernandinho Simonsen, São Paulo, SP, Brasil; 5 Comissão de Educação Continuada (CEC), Sociedade Brasileira de Coluna, São Paulo, SP, Brasil

**Keywords:** scoliosis, evaluation, diagnosis, quality of life

## Abstract

**Objective** The Body Image Disturbance Questionnaire for Scoliosis (BIDQ-S) for scoliosis derives from the Body Image Disturbance Questionnaire (BIDQ) with specific adaptation for scoliosis patients. Despite its significance and applicability, this instrument has never been translated into Brazilian Portuguese. The current study aimed to translate, transculturally adapt, and validate the BIDQ-S into Brazilian Portuguese.

**Methods** BIDQ-S was translated and culturally adapted into Brazilian Portuguese using the American Association of Orthopedic Surgeons (AAOS) criteria. The questionnaire validation relied on internal consistency and comparison with the Cobb angle, Pediatric Quality of Life Inventory (PedsQL), and Scoliosis Research Society (SRS-22). The Brazilian version (BR-)BIDQ-S validation occurred in a sample of 35 adolescents with scoliosis waiting for specialized treatment.

**Results** Internal consistency of the BR-BIDQ-S was 0.899 according to the Cronbach's index (i.e., virtually perfect). Although BR-BIDQ-S did not correlate with the Cobb angle, it presented correlations with the Physical, Emotional, and Social domains from the PedsQL and the Function/Activity domain from the SRS-22.

**Conclusion** BR-BIDQ-S was reliable in evaluating the body image of adolescents with scoliosis, presenting an internal consistency of 0,899 (virtually perfect). Moreover, similar to the original instrument, it correlated with PedsQL and SRS-22.

## Introduction

Scoliosis is a lateral curvature of the spine greater than 10° associated with a rotational component, while spinal asymmetry refers to deviations lower than 10°. 1 Scoliosis classification relies on etiology, curvature location, age of onset, or curvature. 1 Secondary scoliosis or scoliosis of known etiology represents around 20% of all causes, while idiopathic scoliosis is the most common clinical type. 2 3 Idiopathic scoliosis classification may also rely on age as infantile (0–3 years old), juvenile (4–9 years old), and adolescent (over 10 years old). 2

It is worth highlighting that scoliosis does not change the appearance of the back alone but may extend to the anterior region of the trunk, scapulae (shoulder imbalance or elevation), and pelvic and hip tilt. 3 Tones et al. 4 concluded that adolescents with scoliosis can suffer a significant psychosocial impact, especially when presenting prior associated emotional factors. Among psychosocial disorders, concern about body image has become a growing problem impacting the lives and mental health of adolescents. 4 Schwieger et al. 5 analyzed patients from The Bracing in Adolescent Idiopathic Scoliosis Trial (BrAIST) and reported that those with a Cobb angle ≥ 40° had worse scores on the Spinal Appearance Questionnaire (SAQ) and the Pediatric Quality of Life Inventory (PedsQOL) 4.0 Generic Scales even after 2 years of treatment follow-up. These scores reflect a significant loss of self-esteem and quality of life.

The BIDQ-S questionnaire originated from the Body Image Disturbance Questionnaire (BIDQ) proposed by Cash et al. 6 BIDQ-S is an adapted and validated BIQS version by Auerbach et al. 7 to assess body image disorders, especially in scoliosis patients. The version validated by Auerbach et al. 7 is in English, with translations into Korean, German, Turkish, and simplified Chinese. However, there is no validated version in Brazilian Portuguese. Therefore, considering the applicability and significance of this questionnaire, this study aimed to translate the BIDQ-S into Brazilian Portuguese, culturally adapt it, and validate it in this language.

## Casuistry and Methods

This study transcultural adapted and validated BIDQ-S into Brazilian Portuguese using the translation and transcultural adaptation criteria proposed by Beaton et al. 8 (2007and recommended by the American Association of Orthopedic Surgeons (AAOS). 7 After translation, the instrument was validated using a generic questionnaire, a quality of life-specific questionnaire, and the relationship with the Cobb angle. 1 2 3 The study occurred from September 2021 to June 2022 and is part of a larger project approved by the institutional Research Ethics Committee under number CAAE 27816320.0000.5520. All subjects agreeing to participate in the study and their guardians signed the Free and Informed Consent (ICF) and Assent forms.

We performed all stages of translation and cross-cultural validation proposed by AAOS. 8 In the first stage, three bilingual Portuguese-English authors experienced in medicine freely translated (BIDQ-S), 7 from English to Brazilian Portuguese. The second stage consisted of translation harmonization and synthesis. The third stage was an adaptation based on the opinion of three experts in spine who were fluent in English and evaluated the harmonized version. The final version was the result of the second harmonization based on experts' suggestions.

The final version of the BIDQ-S for Brazilian Portuguese underwent a pre-test for cross-cultural adaptation with ten adolescents reporting a complete and sufficient understanding of the instrument. Next, the validation of this version employed 35 patients with adolescent idiopathic scoliosis who were on the waiting list for treatment, using data obtained directly from managers of the Unified Health System (SUS, for its acronym in Portuguese). All patients' contact occurred by letters, telephone calls, electronic means, through patient associations, or in person by the study team.

The research subjects were from the Hospital's Orthopedics Service. The study included all subjects identified as having adolescent idiopathic scoliosis aged from 10 to 18 years old who were awaiting treatment at a service with potential access. Patients with non-idiopathic scoliosis (congenital or neuromuscular), scoliosis lower than 10 degrees, or waiting for a second spinal procedure were excluded.

### Sociodemographic Questionnaire and Radiological Information

The applied sociodemographic and clinical questionnaire asked about age, gender, self-declared ethnicity/race, weight, height, body mass index (BMI), education, and origin. Next, we collected information regarding illness duration, associated conditions, development of secondary sexual characteristics, etc. We also analyzed panoramic radiographs of the patient's spine to determine the Cobb angle of the main curvature and, as a result, the degree of scoliosis (ranging from 1 to 3).

### The instrument: The Body Image Disturbance Questionnaire-Scoliosis (BIDQ-S) 6

The questionnaire had seven items. Items 1, 2, 5, 6, and 7 were subdivided into A and B. Items 1A, 2A, 3, 4, 5A, 6A, and 7A were questions with objective answers about image disturbances. Each question had five answer options, ranging from “not worried” to “extremely worried.” Each answer received a score from 1 (“not worried”) to 5 (“extremely worried”). The final BIDQ-S score was the average of the points obtained on these questions. The higher the score, the greater the body image concern/disturbance level. As mentioned, items 1, 2, 5, 6, and 7 had subdivisions into A and B, with B being subjective characterizations (open responses) of the objective response from subdivision A. In this way, the BIDQ-S instrument presented a quantitative and qualitative analysis of the image disturbances in the patient with scoliosis. 7

### Pediatric Quality of Life Inventory (PedsQL) version for adolescent report (13–18 years old) 9 10

PedsQL has several versions, subdivided by age (5–7, 8–12, and 13–18, 18–25 years old) and by patient or parent report (for patients up to 18 years old). The questionnaire consists of four scales: physical dimension, emotional dimension, social dimension, and educational dimension, and the final scores are divided into these dimensions. The answers range from never (0) to virtually always (4), and the scores undergo a reversible transformation in percentages, i.e., 0 = 100, 1 = 75, 2 = 50, 3 = 25, and 4 = 0. Therefore, when calculating the result, the higher the percentage, the better the quality of life. The dimension cannot be computed if more than 50% of items are incomplete. The result from each domain is the average of the items answered, while the final result is the average of the four separate domains. 9 10

### Scoliosis Research Society (SRS-22r) 11 12

The revised version of the SRS-22 consists of 22 questions divided into five domains, i.e., function/activity, pain, personal image/appearance, mental health, and treatment satisfaction. Answers are objective and range from 1 (worst answer) to 5 (best answer). This questionnaire is validated in Brazilian Portuguese. Data analysis evaluates subtotal and mean values from each domain. The percentage of the result is calculated, considering 100% the best possible answer. 11 12

### Data Analysis

We used the Statistical Package for the Social Sciences (SPSS) software version 25.0 for Windows for data tabulation and analysis. Quantitative variables were expressed as mean ± standard deviation or median and interquartile range (IQR). Qualitative variables were shown as simple and relative frequencies. Statistical analysis consisted of independent samples t-tests, Pearson correlation, ANOVA, and Cronbach's alpha. The significance level was p < 0.05.

## Results

Most subjects were female, around 14 years old, mixed race, attending elementary level II, from the state capital, and with an average Cobb angle above 50 degrees ( Table 1 ).

**Table 1:** Sociodemographic of the sample

Characteristics ( *n* = 35)	N (%) or mean (±standard deviation)
Gender	
Female	27 (77.1%)
Male	8 (22.9%)
Age	14.14 (±1.7)
Weight	51.3 (±11.31)
Height	1.62 (±0.10)
Body mass index	19.76 (±3.81)
Race	
Black	9 (25.7%)
Mixed	22 (62.9%)
White	4 (11.4%)
Education	
Fundamental II	24 (68.6%)
High school	11 (31.4%)
Origin	
State capital	18 (51.4%)
Countryside	17 (48.6%)
Cobb angle from the main curvature	55.50 (±21.16)
Cobb angle lower than 20°	2 (6.7%)
Cobb angle ranging from 20° to 49°	6 (20%)
Cobb angle equal or higher than 50°	22 (73.3%)

The BIDQ-S had the worst scores related to questions 1 ,2 ,3, and 7, in that order, demonstrating concern about appearance, difficulty stopping thinking about appearance, sadness associated with scoliosis, and the need to avoid situations because of scoliosis. There was no difference in the responses of girls compared with boys ( Table 2 ). The internal consistency of the BIDQ-S in the Brazilian Portuguese version was 0.899 per Cronbach's alpha coefficient. This internal consistency is virtually perfect according to the Lands and Koch criteria. 13

**Table 2:** Body Image Disturbance Questionnaire for Scoliosis (BIDQ-S) scores per questions and participants' genders

BIDQ-S	Total	Female	Male	*p*
BIDQ-S – Question 1	2.66 (±1.21)	2.70 (±1.17)	2.50 (±1.41)	0.718
BIDQ-S – Question 2	2.40 (±1.14)	2.33 (±1.11)	2.63 (±1.30)	0.578
BIDQ-S – Question 3	2.43 (±1.17)	2.48 (±1.15)	2.25 (±1.28)	0.656
BIDQ-S – Question 4	1.51 (±0.74)	1.48 (±0.70)	1.63 (±0.92)	0.691
BIDQ-S – Question 5	1.54 (±1.09)	1.63 (±1.18)	1.25 (±0.71)	0.275
BIDQ-S – Question 6	1.63 (±1.06)	1.67 (±1.07)	1.50 (±1.07)	0.706
BIDQ-S – Question 7	2.20 (±1.39)	2.30 (±1.43)	1.88 (±1.25)	0.433
BIDQ-S – Total	2.05 (±0.84)	2.08 (±0.85)	1.95 (±0.85)	0.695

There was no significant correlation between BIDQ-S and the Cobb angle (r = 0.312 with p = 0.094) using Pearson's correlation. Regarding health-related quality of life, measured by PedsQL, the lowest scores were in the Emotional Dimension domain. The PedsQL did not correlate with the Cobb angle (r = -0.259 with p 0.168); there was a correlation between the BIDQ-S and the Physical Dimension, Emotional Dimension, and Social Dimension domains of the PedsQL ( Table 3 ).

**Table 3:** Pediatric Quality of Life Inventory (PedsQL) score and its correlation with the Body Image Disturbance Questionnaire for Scoliosis (BIDQ-S) and Cobb angle

PedsQL domains	PedsQL ( *n* = 35)	BIDQ-S versus PedsQL ( *n* = 35)	BIDQ-S versus Cobb ( *n* = 35)
Physical dimension	70,09 (±22,67)	−0,666 ( *p* < 0,001)	−0,300 ( *p* = 0,107)
Emotional dimension	64,71 (±24,37)	−0,672 ( *p* < 0,001)	−0,060 ( *p* = 0,752)
Social dimension	82,43 (±25,30)	−0,741 ( *p* < 0,001)	−0,261 ( *p* = 0,163)
Educational dimension	69,42 (±12,41)	−0,245 ( *p* = 0,156)	−0,053 ( *p* = 0,779)
Total PedsQL score	71,67 (±16,68)	−0,798 ( *p* < 0,001)	−0,259 ( *p* = 0,168)

Questionnaire comparison used the Pearson correlation.

There was a significant correlation between the BIDQ-S and the SRS-22 in the function/activity dimension. The SRS-22 was also related to the Cobb angle in the function/activity and pain dimensions through Pearson correlation ( Table 4 ).

**Table 4:** Scoliosis Research Society (SRS-22) score and its correlation with the Body Image Disturbance Questionnaire for Scoliosis (BIDQ-S) and Cobb angle

SRS-22 domains	SRS-22 ( *n* = 27)	BIDQ-S ( *n* = 35)	Cobb ( *n* = 30)
Function/activity dimension	3.57 (±0.78)	−0.484 ( *p* = 0.011)	−0.434 ( *p* = 0.039)
Pain dimension	3.63 (±0.96)	−0.234 ( *p* = 0.240)	−0.431 ( *p* = 0.040)
Personal image/appearance dimension	3.27 (±0.68)	−0.022 ( *p* = 0.914)	−0.305 ( *p* = 0.156)
Mental health dimension	3.53 (±0.80)	−0.303 ( *p* = 0.125)	−0.118 ( *p* = 0.593)
Treatment satisfaction dimension	3.76 (±1.08)	−0.067 ( *p* = 0.738)	−0.094 ( *p* = 0.670)
SRS-22 in percentage	70.37 (±11.35)	-0.342 ( *p* = 0.081)	−0.467 ( *p* = 0.025)

Questionnaire comparison used the Pearson correlation.

## Discussion

The Brazilian Portuguese version of the BIDQ-S had an internal consistency of 0.899 according to Cronbach's alpha coefficient, considered virtually perfect. 13 The worst scores were in the domains concerned with appearance, difficulty stopping thinking about appearance, sadness associated with scoliosis, and the need to avoid situations due to scoliosis. Despite not correlating with the Cobb angle, the BIDQ-S questionnaire significantly correlated with the participants' health-related quality of life in the Physical Dimension, Emotional Dimension, and Social Dimension domains of the PedsQL. The BIDQ-S correlated only with the Function/Activity Dimension of the SRS-22.

Our internal consistency is similar to that obtained in equivalent studies described previously and to the original BIDQ and BIDQ-s versions, confirming the validity of the Brazilian Portuguese version of the BIDQ-S. The original BIDQ questionnaire, a generic instrument originating a version for scoliosis created by Cash et al., 6 in 2004, presented a Cronbach's alpha coefficient of 0.89. 13 The Australian version of the BIDQ presented a Cronbach's alpha of 0.92. 14 The adapted version and validated for scoliosis (BIDQ-S), presented by Auerbach et al., 7 2014, had a coefficient of 0.82. 7 The validation of the BIDQ-S questionnaire in its Korean, Turkish, German, and Simplified Chinese had Cronbach's alpha of 0.88, 15 0.88, 16 , 0.87, 17 , and 0.877 17 , respectively.

Our study did not find a significant correlation between the Brazilian Portuguese version of the BIDQ-S and the Cobb angle, even when analyzing patients with and without surgical indication as subgroups. The BIDQ-S validation study from Auerbach et al. 6 ( *N* = 49) also found no correlation between the Cobb angle of the main curvature and the BIDQ-S questionnaire scores. The validation study of the BIDQ-S in its Simplified Chinese version, by Bao et al. apud Wetterkamp et al. 17 ( *N* = 100) also did not observe a significant correlation between the BIDQ-S questionnaire and the Cobb angle. 17 The same study, in a subanalysis, grouped patients with Cobb ≥ 40° and < 40°, observing that those with a Cobb angle ≥ 40° had a higher (worse) C-BIDQ-S result than subjects with a Cobb <40°. 17 The Korean validation study by Bae et al. 15 ( *N* = 113) indicated that patients with larger Cobb angles tend to present worse body image. 15 The German validation study from Wetterkamp et al. 17 ncluding 259 patients was the single one with a significant correlation between the Cobb angle and G-BIDQ-S. 17 Wetterkamp et al *.* 17 hypothesized that the study by Auerbach et al. 7 did not find a significant correlation between the Cobb angle and the BIDQ-S due to a smaller sample size. 17 This can be extrapolated to our study, raising the possibility of a lack of correlation due to the small sample size.

The mean BIDQ-S score in our study was 2.05 (± 0.84), and it was calculated by the average points from questions 1 to 7, ranging from 1 (best possible scenario) to 5 (worst possible scenario). Compared with other studies, the German version had a mean score of 2.04 (± 0.76), including 2.26 ± 0.84 in the surgical subgroup and 1.83 ± 0.60 in the non-surgical subgroup. 17 For the Korean version, the mean score was 2.4 ± 0.8, with the score from the brace user subgroup being significantly higher in comparison with the observation subgroup; moreover, the subgroup requiring surgery had a significantly higher score than the brace subgroup. 15 The mean score of the original validation study from Auerbach et al. 7 was 1.50 ± 0.49, with significant differences between the surgical (1.57), non-surgical (1.45), and control (1.06) subgroups. Our study found the highest BIDQ-S scores in questions 1, 2, and 3, respectively, consistent with the Turkish validation study, with an overall mean value of 2.03 and the highest scores in questions 1 (2.93 ± 1.267), 2 (2.40 ± 1.07), and 3 (2.11 ± 1.12). 16

Our study showed a significant negative correlation between BIDQ-S and SRS-22 in the function/activity dimension. When correlating BIDQ-S with SRS-22, the German validation study found a moderate negative correlation, specifically in the personal image/appearance domain from SRS-22 (−0.74). 17 The Korean version of the BIDQ-S found a significant correlation only for the appearance domain of SRS-22 (p < 0.001, r = −651). 15 Auerbach et al. 7 also found a significant correlation between BIDQ-S and SRS-22 since BIDQ-S correlated with the total SRS-22 (−0.72), the activity (−0.53), pain (−0.53), image (−0.60), and mental (−0.50) domains scores, all with p < 0.001. We believe cultural differences may explain the inconsistency in the correlation between BIDQ-S and SRS-22 in our study and the remaining literature.

Our study found a significant negative correlation between BIDQ-S and PedsQL in the physical, emotional, and social dimensions. Bauer et al. 18 found a strong relationship between BIDQ-S, SRS-22, and PedsQL questionnaires. Their study also highlighted that the mental health domain of SRS-22r correlated well with the emotional domain of PedsQL, suggesting that mental health issues interfere more with personal image than the level of deformity alone. 18 Therefore, these authors emphasize that they chose to apply the BIDQ-S as a body image questionnaire in patients with idiopathic scoliosis as it has a good correlation with other existing questionnaires and fewer questions, consequently requiring a lower application time. 18

Our study has some limitations. The number of patients in our sample, although similar to the one in the original version, may have been relatively small to provide power to all analyses. Our patients were recruited from a clinical-surgical outpatient clinic; therefore, most underwent surgery as the most recommended treatment and usually presented larger Cobb angles. As it occurred in a specialized center for scoliosis treatment, our results may not reflect the same characteristics of the scoliosis population, reducing the external validity of the study. Most of our sample consisted of girls and, although there were no significant differences between genders, this may have given some bias to the work. Despite these limitations, we could validate BIDQ-S in its Brazilian Portuguese version and carry out fundamental secondary analyses confirmed in similar studies.

## Conclusion

Our study demonstrated that the Brazilian Portuguese version of the BIDQ-S (BR-BIDQ-S) is a reliable instrument for assessing adolescent body image, with an internal validity of 0.899 (virtually perfect), which is similar to the internal consistency of the original instrument. Furthermore, BR-BIDQ-S correlated with the quality of life of adolescents measured by PedsQL and with the Function/Activity Domain of SRS-22, reinforcing its validity.

**Appendix 1:** Original and Brazilian Portuguese versions Body Image Disturbance Questionnaire for Scoliosis (BIDQ-S)

English	Brazilian Portuguese
1A. Are you worried about the appearance of your back shape? 1. Not at all worried 2. Somewhat worried 3. Moderately worried 4. Very worried 5. Extremely worried	1A. 1A Você está preocupado com a aparência da forma de suas costas? 1. Não 2. Um pouco 3. Moderadamente 4. Muito 5. Extremamente
1B. What are these concerns? 1. My shoulders are uneven (one is higher or lower than the other) 2. My shoulder blade sticks out 3. My chest is asymmetric from the front (one side looks higher or lower than the other side) 4. My hips are asymmetric (one hip is higher or lower than the other) 5. My rib bump	1B. Quais são essas preocupações? 1. Meus ombros são desalinhados (um é mais alto ou mais baixo que o outro) 2. Minha escápula fica aparente 3. Meu tórax é assimétrico olhando de frente (um lado é mais alto ou mais baixo que o outro) 4. Meus quadris são assimétricos (um lado é mais alto ou baixo que o outro) 5. A deformidade das minhas costelas
2A. If you are at least somewhat concerned or worried, do these concerns/worries preoccupy you? That is, you think about them a lot and they're hard to stop thinking about? (Circle the best answer) 1. Not at all preoccupied (I do not think about them) 2. Somewhat preoccupied (I think about them from time to time) 3. Moderately preoccupied (I think about them a moderate amount) 4. Very preoccupied (I think about them a lot) 5. Extremely preoccupied (I think about them constantly)	2A. Se você está ao menos um pouco preocupado, você pensa muito sobre o assunto e é difícil parar de pensar sobre isso? 1. Não (não penso sobre) 2. Um pouco (penso de vez em quando) 3. Moderadamente (penso em uma quantidade moderada) 4. Muito (penso muito) 5. Extremamente (penso constantemente)
2B. How do your concerns about the way your back looks affect your life? For example, some kids say that they avoid swimming because they are embarrassed to show their back.	2B. Como as preocupações sabre a aparência de suas costas afetam sua vida? Por exemplo, algumas crianças falam que evitam nadar porque têm vergonha de mostrar suas costas.
3. Has the way your back looks caused you to feel upset? How much? 1. Not upset at all 2. Mild (a little bit upset) 3. Moderate (Somewhat upset) 4. Severe, and very disturbing (very upset) 5. Extreme, and disabling (extremely upset)	3. A forma de suas costas já fez você se sentir triste? Quanto? 1. Não 2. Um pouco triste 3. Moderadamente triste 4. Muito triste, muito perturbador 5. Extremamente triste, incapacitante
4. Have your worries about how your back looks caused you any problems at school, at your job, or with your friends and family? How much? 1. No problems 2. A few problems, but overall I can do what I need to do, and my performance is not affected 3. Several problems, but I can cope with them; problems are still manageable 4. A lot of problems that limit what I can do; problems cause a lot of limitations 5. Extreme problems that keep me from doing almost everything I want or need to do	4. As suas preocupações sobre a forma de suas costas causaram algum problema na escola, trabalho ou com seus amigos e família? Quanto? 1. Não 2. Alguns problemas, mas no geral eu posso fazer o que preciso fazer, e minha performance não é afetada 3. Muitos problemas, mas posso lidar com isso, problemas que consigo manejar 4. Vários problemas que limitam o que posso fazer, problemas que causam muitas limitações 5. Problemas extremos que me impedem de fazer quase tudo que quero ou preciso fazer
5A. Has your back shape caused problems with your friends, family members, or dating? How much? 1. Never 2. Occasionally 3. Sometimes 4. A lot 5. All the time	5A. A forma de suas costas causou problemas com seus amigos, membros da família ou relacionamentos? Quanto? 1. Nunca 2. Ocasionalmente 3. Às vezes 4. Muito 5. O tempo todo
5B. If so, how?	5B. Se sim, como?
6A. Has your back shape caused problems with your schoolwork, your job, or your ability to do other things that are important to you (e.g., play sports, be social with your friends)? How much? 1. Never 2. Occasionally 3. Sometimes 4. A lot 5. All the time	6A A forma de suas costas já causou problemas com trabalhos de escola, trabalho ou sua habilidade de fazer outras coisas que são importantes para você? (ex: esportes, sair com os amigos)? Quanto? 1. Nunca 2. Ocasionalmente 3. Às vezes 4. Muito 5. O tempo todo
6B. If so, how?	6B. Se sim, como?
7A. Do you ever avoid things because of your back shape? How often? (Circle the best answer) 1. Never 2. Occasionally 3. Sometimes 4. A lot 5. All the time	7A. Você já evitou situações por causa da forma de suas costas? Quão frequentemente? 1. Nunca 2. Ocasionalmente 3. Às vezes 4. Muito 5. O tempo todo
7B. If so, how?	7B. Se sim, como?
