# Supplementary material for: Translation, Transcultural Adaptation, and Validation of the Brazilian Portuguese Version of the Body Image Disturbance Questionnaire for Scoliosis (BR-BIDQ-S)
Source: Rev Bras Ortop (Sao Paulo). 2024 Apr 10;59(2):e220–7. doi: 10.1055/s-0044-1785463 (PMC11006525; doi:10.1055/s-0044-1785463)
Supplement: Related texts

# Research article: Tradução, adaptação transcultural e validação da versão para o português brasileiro do *Body Image Disturbance Questionnaire* para escoliose (BR-BIDQ-S)

**Keywords:** escoliose, avaliação, diagnóstico, qualidade de vida

## Abstract

**Objetivo** O questionário BIDQ-S para escoliose se originou a partir do Body Image Disturbance Questionnaire (BIDQ) e representa a versão adaptada e validada especificamente em pacientes com escoliose. Este instrumento, a despeito da sua aplicabilidade e importância, ainda não tem versão para uso no Brasil. O presente estudo teve como objetivo traduzir, adaptar transcuturalmente e validar o BIDQ-S para o português brasileiro.

**Método** Foi realizado estudo de tradução adaptação transcultural e validação do instrumento Body Image Disturbance Questionnaire-Scoliosis version (BIDQ-S) para o português brasileiro, utilizando os critérios recomendados pela American Association of Orthopedic Surgeon (AAOS). A validação do instrumento foi realizada por meio de consistência interna e confrontando-se com ângulo de Cobb, Pediatric Quality of Life Inventory (PedsQL) e Scoliosis Research Society (SRS-22). O BR-BIDQ-S (versão brasileira) foi validado em um grupo de 35 adolescentes portadores de escoliose idiopática do adolescente que se encontravam na fila de espera para tratamento.

**Resultados** A consistência interna do Br-BIDQ-S foi de 0,899 medido pelo coeficiente Alfa de Cronbach (considerada quase perfeita). Não houve correlação com o ângulo de Cobb, contudo o Br-BIDQ-S teve correlação com os domínios Dimensão Física, Dimensão Emocional e Dimensão Social do PedsQL e também com a Dimensão Função/Atividade do SRS-22.

**Conclusão** O Br-BIDQ-S mostrou-se confiável para avaliar a imagem corporal de adolescentes com escoliose, com validade interna de 0,899 (considerada quase perfeita), similar àquela do instrumento original, havendo correlação com o PedsQL e com o SRS-22.

## Introdução

Escoliose se define como uma curvatura lateral na coluna maior que 10°, associada a componente rotatório, sendo que desvios menores que 10° são chamados de assimetria espinhal. 1 A escoliose pode ser classificada por etiologia, localização da curva, idade de início ou tipo de curva. 1 Escoliose secundária ou de etiologia conhecida representa cerca de 20% de todas as causas, enquanto que a escoliose idiopática é o tipo clínico mais comum. 2 3 A escoliose idiopática ainda pode ser dividida de acordo com a idade em infantil (0–3 anos), juvenil (4–9 anos) e do adolescente (maior que 10 anos). 2

É importante ressaltar que a escoliose não causa alterações na aparência apenas das costas, a deformidade pode se estender para a região anterior do tronco, escápulas (desequilíbrio nos ombros ou elevação dos ombros), obliquidade pélvica e quadris. 3 Tones et al. 4 concluíram que adolescentes com escoliose podem apresentar impacto psicossocial importante, principalmente quando já existem fatores emocionais associados. Dentre os distúrbios psicossociais, a preocupação com a imagem corporal tem se tornado problema crescente com impactos na vida e na saúde mental dos adolescentes. 4 Schwieger et al., 5 ao analisarem pacientes do The Bracing in Adolescent Idiopathic Scoliosis Trial (BrAIST), relataram que pacientes com ângulo de Cobb de ≥ 40° apresentava piores escores nos questionários Spinal Appearance Questionnaire (SAQ), e no Pediatric Quality of Life Inventory (PedsQOL) 4.0 Generic Scales, mesmo após 2 anos de follow-up no tratamento, refletindo importante perda de autoestima e de qualidade de vida.

O questionário BIDQ-S se originou a partir do Body Image Disturbance Questionnaire (BIDQ) proposto por Cash et al. 6 *O BIDQ-S* nada mais é do que a versão adaptada e validada por Auerbach et al. 7 para avaliar distúrbios de imagem corporal especificamente em pacientes com escoliose. A versão validada por Auerbach et al 7 está em língua inglesa e, ao longo da concepção desse trabalho foram encontradas versões do BIDQ-S em coreano, alemão, turco, e chinês simplificado. Entretanto, não foi encontrada versão validada em português brasileiro. Dessa forma, considerando a aplicabilidade e importância deste questionário, este estudo teve como objetivo traduzir o BIDQ-S para o português brasileiro, a fim de adaptar culturalmente e validar a versão nesta língua.

## Casuística e Métodos

Foi realizado estudo de tradução adaptação transcultural e validação do instrumento Body Image Disturbance Questionnaire-Scoliosis version (BIDQ-S) para o português brasileiro, utilizando os critérios de tradução e adaptação transcultural propostos por Beaton et al. 8 (2007) e recomendados pela American Association of Orthopedic Surgeon (AAOS). 7 Depois da tradução, foi realizada validação do instrumento utilizando-se um questionário genérico e outro específico para qualidade de vida, além da relação do instrumento com o ângulo de Cobb. 1 2 3 O estudo foi realizado no período de setembro de 2021 a junho de 2022 e faz parte de um projeto maior, aprovado pelo Comitê de Ética em Pesquisa institucional sob número CAAE 27816320.0000.5520. Todos os sujeitos que aceitaram participar do estudo e seus responsáveis assinaram o Termo de consentimento Livre e Esclarecido (TCLE) e o termo de Assentimento.

Foram realizadas todas as etapas de tradução e validação transcultural propostas pela AAOS. 8 Na primeira etapa, três autores bilíngues português-inglês, com experiência na área de medicina, fizeram a tradução livre do Body Image Disturbance Questionnaire-Scoliosis version (BIDQ-S), 7 originalmente em inglês, para Português Brasileiro. Na segunda etapa foi realizada harmonização e síntese da tradução. A terceira etapa foi adaptada para o estudo utilizando-se a opinião de três experts na área de coluna com fluência em inglês para avaliação da versão harmonizada. A versão final foi obtida após a segunda harmonização, a partir de sugestões dos experts.

A versão final do BIDQ-S para o português brasileiro sofreu um pré-teste para adaptação transcultural com um grupo de 10 adolescentes que relataram compreensão completa e suficiente do instrumento. Esta versão foi então utilizada para validação em um grupo de 35 adolescentes portadores de escoliose idiopática do adolescente que se encontravam na fila de espera para tratamento, por meio de dados obtidos diretamente de gestores do Sistema Único de Saúde (SUS). Todos os pacientes foram contatados por carta, telefone, meios eletrônicos, por intermédio de associações de pacientes ou pessoalmente pela equipe do estudo.

Os sujeitos da pesquisa foram recrutados no Serviço de Ortopedia do Hospital. Foram incluídos no estudo todos os sujeitos identificados como portadores de escoliose idiopática do adolescente entre 10 e 18 anos que aguardam tratamento na fila de espera do Serviço cujo acesso tenha sido possível. Foram excluídos pacientes que tenham escoliose não idiopática (congênita ou neuromuscular), menor que 10 graus, ou em espera de segundo procedimento na coluna vertebral.

## Questionário Sociodemográfico e Informações de Radiografia

Foi aplicado um questionário sociodemográfico e clínico constituído de perguntas como idade, sexo, etnia/raça autodeclarada, peso, altura, IMC, escolaridade, procedência. Após a coleta dessas informações, foram coletadas informações constantes a respeito do tempo de doença, condições associadas, desenvolvimento de caracteres sexuais secundários, etc. Também foi analisada a radiografia panorâmica da coluna vertebral dos pacientes, a fim de determinar o ângulo de Cobb da curva principal e, consequentemente, o grau da escoliose (1–3).

## O instrumento: The Body Image Disturbance Questionnaire-Scoliosis (BIDQ-S) 6

O questionário é composto por 7 itens, sendo que os itens 1, 2 e 5–7 são subdivididos em A e B. Os itens 1A, 2A, 3, 4, 5A, 6A, e 7A correspondem a perguntas com respostas objetivas sobre distúrbios de imagem. Essas perguntas têm cinco opções de respostas cada, que variam de: “não estou preocupado” até “extremamente preocupado.” Para cada uma das respostas é atribuída uma pontuação de 1 (“não estou preocupado”) a 5 (“extremamente preocupado”). O escore final do BIDQ-S é conseguido através da média das pontuações obtidas nessas perguntas. Quanto maior a pontuação, maior o nível de preocupação/ distúrbio de imagem corporal. Os itens 1, 2, e 5–7 possuem subdivisões em A e B, sendo o item B caracterizações subjetivas (respostas abertas) da resposta objetiva obtida na subdivisão A. Dessa forma, o instrumento BIDQ-S apresenta uma análise quantitativa e qualitativa dos distúrbios de imagem no paciente com escoliose. 7

## Pediatric Quality of Life Inventory (PedsQL) versão relato do adolescente (13–18 anos) 9 10

O Questionário Pediátrico sobre Qualidade de Vida PedsQL tem diversas versões, subdivididas por idade (5–7, 8–12 e 13–18, 18–25 anos) e por relato dos pacientes ou relato dos pais sobre seus filhos (no caso de pacientes até 18 anos). O questionário também é agrupado em quatro escalas: dimensão física, dimensão emocional, dimensão social e dimensão escolar, sendo que os escores de pontuação final também são divididos nessas dimensões. As respostas variam de nunca (0) a quase sempre (4), que serão transformadas de forma reversa em porcentagem, ou seja, 0 = 100, 1 = 75, 2 = 50, 3 = 25, 4 = 0. Dessa forma, ao calcular o resultado, quanto maior a porcentagem, melhor a qualidade de vida. Se mais de 50% dos itens não estiverem preenchidos, a dimensão não deve ser computada. Para fazer o resultado de cada domínio, é feita a média dos itens respondidos em cada respectivo domínio; o resultado final é a média dos quatro domínios em separado. 9 10

## Scoliosis Research Society (SRS-22r) 11 12

A versão revisada do SRS-22 é formada por 22 questões, divididas em cinco domínios, sendo eles função/atividade, dor, imagem pessoal/aparência, saúde mental e satisfação com o tratamento. As respostas são objetivas e variam de 1 (pior resposta) a 5 (melhor resposta). É um questionário já validado em português brasileiro. Para análise de dados, foram avaliados subtotais e médias de cada domínio, bem como foi calculada a porcentagem do resultado, considerando 100% a melhor resposta possível. 11 12

## Análise dos Dados

Para tabulação e análise dos dados foi utilizado o software SPSS ( *Statistical Package for the Social Sciences* ) versão 25.0 para Windows. As variáveis quantitativas foram expressas através de média ± desvio padrão ou mediana e amplitude interquartil (AIQ). As variáveis qualitativas foram expressas através de frequências simples e relativas. Para análise estatística também foram utilizados os testes T de amostras independentes, correlação de Pearson, ANOVA e Alfa de Cronbach. Foi considerado nível de significância *p* < 0.05.

## Resultados

A maioria dos sujeitos foi do sexo feminino ao redor de 14 anos, pardos, cursando o nível educacional fundamental II, procedentes da capital do estado e com média do ângulo de Cobb acima de 50 graus ( Tabela 1 ).

**Tabela 1:** Distribuição sociodemográficos dos respondentes

Características ( *n* = 35)	N (%) ou média (±desvio-padrão)
Sexo	
Meninas	27 (77,1%)
Meninos	8 (22,9%)
Idade	14,14 (±1,7)
Peso	51,3 (±11,31)
Altura	1,62 (±0,10)
IMC	19,76 (±3,81)
Raça	
Negro	9 (25,7%)
Pardo	22 (62,9%)
Branco	4 (11,4%)
Escolaridade	
Fundamental II	24 (68,6%)
Médio	11 (31,4%)
Procedência	
Capital	18 (51,4%)
Interior	17 (48,6%)
Ângulo de Cobb da curva principal	55,50 (±21,16)
Ângulo de Cobb abaixo de 20°	2 (6,7%)
Ângulo de Cobb entre 20° e 49°	6 (20%)
Ângulo de Cobb maior ou igual a 50°	22 (73,3%)

O BIDQ-S teve os piores escores relacionados às questões 1,2,3, e 7 nesta ordem. Demonstrando preocupação com aparência, dificuldade de parar de pensar sobre a aparência, sentimento de tristeza associado à escoliose, e necessidade de evitar situações por causa da escoliose. Não houve diferença nas respostas das meninas em comparação com os meninos ( Tabela 2 ). A consistência interna do BIDQ-S na versão do português brasileiro foi de 0,899 medido pelo coeficiente Alfa de Cronbach. Esta consistência interna é considerada quase perfeita pelos critérios de Lands e Koch. 13

**Tabela 2:** Escores do BIDQ-S dividido de acordo com as questões e com o sexo dos respondentes

BIDQ-S	Total	Meninas	Meninos	*p*
BIDQ-S – Questão 1	2,66 (±1,21)	2,70 (±1,17)	2,50 (±1,41)	0,718
BIDQ-S – Questão 2	2,40 (±1,14)	2,33 (±1,11)	2,63 (±1,30)	0,578
BIDQ-S – Questão 3	2,43 (±1,17)	2,48 (±1,15)	2,25 (±1,28)	0,656
BIDQ-S – Questão 4	1,51 (±0,74)	1,48 (±0,70)	1,63 (±0,92)	0,691
BIDQ-S – Questão 5	1,54 (±1,09)	1,63 (±1,18)	1,25 (±0,71)	0,275
BIDQ-S – Questão 6	1,63 (±1,06)	1,67 (±1,07)	1,50 (±1,07)	0,706
BIDQ-S – Questão 7	2,20 (±1,39)	2,30 (±1,43)	1,88 (±1,25)	0,433
BIDQ-S - Total	2,05 (±0,84)	2,08 (±0,85)	1,95 (±0,85)	0,695

Não houve correlação significante entre BIDQ-S e ângulo de Cobb (r = 0,312 com *p* = 0,094) através da correlação de Pearson. No que se refere à qualidade de vida relacionada à saúde, medida pelo PedsQL, observou-se os menores escores no domínio Dimensão emocional. O PedsQL não teve correlação com o ângulo de Cobb (r = −0,259 com *p* = 0,168); houve correlação do BIDQ-S com os domínios Dimensão física, Dimensão emocional e Dimensão social do PedsQL ( Tabela 3 ).

**Tabela 3:** Escores do PedsQL e sua correlação com BIDQ-S e com ângulo de Cobb

Domínios do PedsQL	PedsQL ( *n* = 35)	BIDQ-S versus PedsQL ( *n* = 35)	BIDQ-S versus Cobb ( *n* = 35)
Dimensão física	70,09 (±22,67)	−0,666 ( *p* < 0,001)	−0,300 ( *p* = 0,107)
Dimensão emocional	64,71 (±24,37)	−0,672 ( *p* < 0,001)	−0,060 ( *p* = 0,752)
Dimensão social	82,43 (±25,30)	−0,741 ( *p* < 0,001)	−0,261 ( *p* = 0,163)
Dimensão escolar	69,42 (±12,41)	−0,245 ( *p* = 0,156)	−0,053 ( *p* = 0,779)
Total PedsQL	71,67 (±16,68)	−0,798 ( *p* < 0,001)	−0,259 ( *p* = 0,168)

Correlação de Pearson foi utilizada para a comparação dos questionários.

Houve correlação significante entre o BIDQ-S e o SRS-22 na dimensão função/atividade. O SRS-22 também se relacionou ao ângulo de Cobb nas dimensões função/atividade e dor, através da correlação de Pearson Tabela 4 .

**Tabela 4:** Escores do SRS-22 e sua correlação com BIDQ-S e com ângulo de Cobb

Domínios do SRS-22	SRS-22 ( *n* = 27)	BIDQ-S ( *n* = 35)	Cobb ( *n* = 30)
Dimensão função/atividade	3,57 (±0,78)	−0,484 ( *p* = 0,011)	−0,434 ( *p* = 0,039)
Dimensão dor	3,63 (±0,96)	−0,234 ( *p* = 0,240)	−0,431 ( *p* = 0,040)
Dimensão imagem pessoal/ aparência	3,27 (±0,68)	−0,022 ( *p* = 0,914)	−0,305 ( *p* = 0,156)
Dimensão saúde mental	3,53 (±0,80)	−0,303 ( *p* = 0,125)	−0,118 ( *p* = 0,593)
Dimensão satisfação com o tratamento	3,76 (±1,08)	−0,067 ( *p* = 0,738)	−0,094 ( *p* = 0,670)
Porcentagem SRS-22	70,37 (±11,35)	−0,342 ( *p* = 0,081)	−0,467 ( *p* = 0,025)

Correlação de Pearson foi utilizada para a comparação dos questionários.

## Discussão

A versão para o português brasileiro do BIDQ-S teve consistência interna de 0,899 pelo coeficiente alfa de Cronbach, considerada quase perfeita. 13 Os piores escores estiveram relacionados aos domínios preocupação com aparência, dificuldade de parar de pensar sobre a aparência, sentimento de tristeza associado à escoliose, e necessidade de evitar situações por causa da escoliose. A despeito de não ter tido correlação com o ângulo de Cobb, o questionário BIDQ-S teve correlação significante com a qualidade de vida relacionada à saúde dos participantes nos domínios Dimensão física, Dimensão emocional e Dimensão social do PedsQL. O BIDQ-S se correlacionou somente com a Dimensão Função/Atividade do SRS-22.

Nossa consistência interna assemelha-se àquela obtida em trabalhos semelhantes descritos anteriormente e às versões originais do BIDQ e do BIDQ-s, confirmando a validade da versão para o português brasileiro do BIDQ-S. O questionário original BIDQ, instrumento genérico que deu origem à versão para escoliose, criado Cash et al., 6 em 2004, apresentou coeficiente Alfa de Cronbach de 0.89. 13 A versão australiana do BIDQ apresentou Alfa de Cronbach de 0.92. 14 A versão adaptada e validada para escoliose (BIDQ-S), apresentada por Auerbach et al., 7 2014, apresentou coeficiente de 0.82. 7 A validação do questionário BIDQ-S em sua versão coreana apresentou Alfa de Cronbach de 0.88 15 ; a versão turca do BIDQ-S apresentou Alfa de Cronbach de 0.88 16 ; a versão alemã apresentou Alfa de Cronbach de 0.87 17 e a versão em chinês simplificado apresentou Alfa de Cronbach de 0.877. 17

Nosso estudo não encontrou correlação significante entre a versão para o português brasileiro do BIDQ-S e o ângulo de Cobb, mesmo ao analisar em subgrupos pacientes com indicação cirúrgica e sem indicação cirúrgica. O estudo de validação do BIDQ-S, conduzido por Auerbach et al. 6 ( *N* = 49) também não encontrou correlação entre ângulo de Cobb da curva principal e os escores do questionário BIDQ-S. O estudo de validação do BIDQ-S em sua versão chinês simplificado, por Bao et al. apud Wetterkamp et al. 17 ( *N* = 100), também não observou correlação significante entre o questionário BIDQ-S e o ângulo de Cobb. 17 O mesmo estudo, ao fazer análise de subgrupos, agrupados entre pacientes com Cobb ≥ 40° e <40°, observou que pacientes com ângulo de Cobb ≥ 40° tinham um maior (pior) resultado do C-BIDQ-S que os pacientes com Cobb <40°. 17 O estudo de validação coreana, por Bae et al. 15 ( *N* = 113), sinalizou que pacientes com ângulos de Cobb maiores tendem a apresentar impacto negativo na imagem corporal. 15 O estudo de validação alemã, realizado por Wetterkamp et al. 17 Que incluiu 259 pacientes foi o único que demonstrou correlação significante entre o ângulo de Cobb e G-BIDQ-S. 17 Wetterkamp et al *.* 17 levantaram a hipótese de que o estudo por Auerbach et al. 7 não encontrou correlação significante entre o Cobb e o BIDQ-S devido a um tamanho mais reduzido da amostra. 17 Isso pode, potencialmente, ser extrapolado para o presente estudo, sendo levantada a possibilidade de ausência de correlação devido ao tamanho reduzido da amostra.

A média de resultado do BIDQ-S encontrado no presente estudo foi de 2,05 (±0,84), sendo calculado pela média das questões 1 a 7, com scores variando entre 1 (melhor cenário possível) e 5 (pior cenário possível). Quando comparado aos demais estudos, a versão alemã apresentou uma média de resultado de 2.04 (±0.76) sendo 2.26 ± 0.84 no subgrupo cirúrgico e 1.83 ± 0.60 no subgrupo não cirúrgico (17). A versão coreana teve como média de resultado 2.4 ± 0.8, sendo que o acometimento do subgrupo de usuários de colete foi significantemente maior que o do subgrupo observação, e do subgrupo com necessidade cirúrgica sendo significantemente maior que a do subgrupo colete. 15 Já a média do estudo de validação original, apresentado por Auerbach et al., 7 foi de 1.50 ± 0.49, sendo que o escore entre os subgrupos cirúrgico (1.57), não cirúrgico (1.45) e controle (1.06) diferiram significantemente. Nosso estudo encontrou os maiores escores do BIDQ-S nas questões 1, 2 e 3, respectivamente, o que está em consonância com o estudo de validação turco, que obteve média global de 2.03, sendo os maiores escores nas questões 1 (2,93 ± 1,267), 2 (2,40 ± 1,07) e 3 (2,11 ± 1,12). 16

Em nosso estudo, houve correlação negativa significante entre o BIDQ-S e o SRS-22 na dimensão função/atividade. Ao correlacionar o BIDQ-S com o SRS-22, o estudo de validação alemão encontrou moderada correlação negativa, especificamente no domínio imagem pessoal/Aparência do SRS-22 (−0,74). 17 A versão coreana do BIDQ-S encontrou correlação significativa apenas com o domínio aparência do SRS-22 ( *p* < 0.001, r = −651). (15) O estudo por Auerbach et al. 7 também encontrou correlação significativa entre o BIDQ-S e o SRS-22. Sendo que o BIDQ-S se correlacionou com o total do SRS-22 (−0,72), com os domínios atividade (−0,53), dor (−0,53), imagem (−0,60) e domínio mental (−0,50), todos com *p* < 0,001. Acreditamos que diferenças culturais possam explicar a inconsistência de correlação entre o BIDQ-S e o SRS-22 no nosso e nos outros trabalhos da literatura.

Nosso estudo encontrou correlação negativa significativa entre o BIDQ-S e o PedsQL nas dimensões física, emocional e social. Em um estudo realizado por Bauer et al., 18 um dos achados descritos foi a forte relação entre o BIDQ-S e os questionários SRS-22 e PedsQL. Nesse estudo, ainda foi ressaltado que o domínio saúde mental do SRS-22r se correlacionaram bem com o domínio emocional do PedsQL, o que sugeriu que questões relacionadas à saúde mental interferem mais na imagem pessoal que o nível da deformidade isoladamente. 18 Dessa forma, esses autores ressaltam que optaram por aplicar o BIDQ-S como questionário de imagem corporal em pacientes com escoliose idiopática, por ter uma boa correlação com os demais questionários existentes e menos perguntas, exigindo, consequentemente, menos tempo de aplicação. 18

Algumas limitações devem ser consideradas antes da interpretação dos resultados de nosso estudo. O número de pacientes na amostra, embora semelhante à versão original, pode ter sido relativamente pequeno para conferir poder a todas as análises. Nossos pacientes foram recrutados em um ambulatório clínico-cirúrgico, logo, a maioria dos pacientes tinha a cirurgia como tratamento mais indicado, bem como este tipo de amostra tende a apresentar pacientes com ângulo de Cobb maior. Por ter sido realizado em centro especializado para tratamento da escoliose, os resultados do presente estudo podem não refletir as mesmas características populacionais da escoliose, diminuindo a validade externa do estudo. A maior parte da nossa amostra foi constituída de meninas e, apesar de não ter sido observada diferença significativa entre os sexos, isto pode ter conferido algum viés ao trabalho. A despeito destas limitações, foi possível validar o BIDQ-S na sua versão para o português brasileiro e realizar análises secundárias importantes que foram confirmadas em outros estudos semelhantes.

## Conclusão

Nosso estudo demonstrou que a versão do português brasileiro do BIDQ-S (Br-BIDQ-S) é um instrumento confiável para avaliar a imagem corporal de adolescentes, com validade interna de 0,899 (considerada quase perfeita), sendo similar à consistência interna do instrumento original. Além disto, o Br-BIDQ-S se correlacionou com a qualidade de vida dos adolescentes medida pelo PedsQL e com o Domínio Função/Atividade do SRS-22, reforçando a validade do Br-BIDQ-S.

**Anexo 1:** Versão original e em português brasileiro do BIDQ-S

Inglês	Português-BR
1A. Are you worried about the appearance of your back shape? 1. Not at all worried 2. Somewhat worried 3. Moderately worried 4. Very worried 5. Extremely worried	1A. 1A Você está preocupado com a aparência da forma de suas costas? 1. Não 2. Um pouco 3. Moderadamente 4. Muito 5. Extremamente
1B. What are these concerns? 1. My shoulders are uneven (one is higher or lower than the other) 2. My shoulder blade sticks out 3. My chest is asymmetric from the front (one side looks higher or lower than the other side) 4. My hips are asymmetric (one hip is higher or lower than the other) 5. My rib bump	1B. Quais são essas preocupações? 1. Meus ombros são desalinhados (um é mais alto ou mais baixo que o outro) 2. Minha escápula fica aparente 3. Meu tórax é assimétrico olhando de frente (um lado é mais alto ou mais baixo que o outro) 4. Meus quadris são assimétricos (um lado é mais alto ou baixo que o outro) 5. A deformidade das minhas costelas
2A. If you are at least somewhat concerned or worried, do these concerns/worries preoccupy you? That is, you think about them a lot and they're hard to stop thinking about? (Circle the best answer) 1. Not at all preoccupied (I do not think about them) 2. Somewhat preoccupied (I think about them from time to time) 3. Moderately preoccupied (I think about them a moderate amount) 4. Very preoccupied (I think about them a lot) 5. Extremely preoccupied (I think about them constantly)	2A. Se você está ao menos um pouco preocupado, você pensa muito sobre o assunto e é difícil parar de pensar sobre isso? 1. Não (não penso sobre) 2. Um pouco (penso de vez em quando) 3. Moderadamente (penso em uma quantidade moderada) 4. Muito (penso muito) 5. Extremamente (penso constantemente)
2B. How do your concerns about the way your back looks affect your life? For example, some kids say that they avoid swimming because they are embarrassed to show their back.	2B. Como as preocupações sabre a aparência de suas costas afetam sua vida? Por exemplo, algumas crianças falam que evitam nadar porque têm vergonha de mostrar suas costas.
3. Has the way your back looks caused you to feel upset? How much? 1. Not upset at all 2. Mild (a little bit upset) 3. Moderate (Somewhat upset) 4. Severe, and very disturbing (very upset) 5. Extreme, and disabling (extremely upset)	3. A forma de suas costas já fez você se sentir triste? Quanto? 1. Não 2. Um pouco triste 3. Moderadamente triste 4. Muito triste, muito perturbador 5. Extremamente triste, incapacitante
4. Have your worries about how your back looks caused you any problems at school, at your job, or with your friends and family? How much? 1. No problems 2. A few problems, but overall I can do what I need to do, and my performance is not affected 3. Several problems, but I can cope with them; problems are still manageable 4. A lot of problems that limit what I can do; problems cause a lot of limitations 5. Extreme problems that keep me from doing almost everything I want or need to do	4. As suas preocupações sobre a forma de suas costas causaram algum problema na escola, trabalho ou com seus amigos e família? Quanto? 1. Não 2. Alguns problemas, mas no geral eu posso fazer o que preciso fazer, e minha performance não é afetada 3. Muitos problemas, mas posso lidar com isso, problemas que consigo manejar 4. Vários problemas que limitam o que posso fazer, problemas que causam muitas limitações 5. Problemas extremos que me impedem de fazer quase tudo que quero ou preciso fazer
5A. Has your back shape caused problems with your friends, family members, or dating? How much? 1. Never 2. Occasionally 3. Sometimes 4. A lot 5. All the time	5A. A forma de suas costas causou problemas com seus amigos, membros da família ou relacionamentos? Quanto? 1. Nunca 2. Ocasionalmente 3. Às vezes 4. Muito 5. O tempo todo
5B. If so, how?	5B. Se sim, como?
6A. Has your back shape caused problems with your schoolwork, your job, or your ability to do other things that are important to you (e.g., play sports, be social with your friends)? How much? 1. Never 2. Occasionally 3. Sometimes 4. A lot 5. All the time	6A A forma de suas costas já causou problemas com trabalhos de escola, trabalho ou sua habilidade de fazer outras coisas que são importantes para você? (ex: esportes, sair com os amigos)? Quanto? 1. Nunca 2. Ocasionalmente 3. Às vezes 4. Muito 5. O tempo todo
6B. If so, how?	6B. Se sim, como?
7A. Do you ever avoid things because of your back shape? How often? (Circle the best answer) 1. Never 2. Occasionally 3. Sometimes 4. A lot 5. All the time	7A. Você já evitou situações por causa da forma de suas costas? Quão frequentemente? 1. Nunca 2. Ocasionalmente 3. Às vezes 4. Muito 5. O tempo todo
7B. If so, how?	7B. Se sim, como?
